# A Comparative Study of Frequent Pattern Mining with Trajectory Data

**DOI:** 10.3390/s22197608

**Published:** 2022-10-07

**Authors:** Shiting Ding, Zhiheng Li, Kai Zhang, Feng Mao

**Affiliations:** 1 Tsinghua Shenzhen International Graduate School, Tsinghua University, Shenzhen 518055, China; 2 Research Institute of Tsinghua, Pearl River Delta, Guangzhou 510530, China

**Keywords:** data mining, vehicle trajectory, sequential pattern mining, traffic congestion

## Abstract

Sequential pattern mining (SPM) is a major class of data mining topics with a wide range of applications. The continuity and uncertain nature of trajectory data make it distinctively different from typical transactional data, which requires additional data transformation to prepare for SPM. However, little research focuses on comparing the performance of SPM algorithms and their applications in the context of trajectory data. This study selected some representative sequential pattern mining algorithms and evaluated them with various parameters to understand the effect of the involved parameters on their performances. We studied the resultant sequential patterns, runtime, and RAM consumption in the context of the taxi trajectory dataset, the T-drive dataset. It was demonstrated in this work that a method to discretize trajectory data and different SPM algorithms were performed on trajectory databases. The results were visualized on actual Beijing road maps, reflecting traffic congestion conditions. Results demonstrated contiguous constraint-based algorithms could provide a concise representation of output sequences and functions at low min_sup with balanced RAM consumption and execution time. This study can be used as a guide for academics and professionals when determining the most suitable SPM algorithm for applications that involve trajectory data.

## 1. Introduction

Location-detection devices such as GPS and FRID provide the ability to log an object’s travel pattern. Trajectories denote the paths traced by bodies moving in space over time [1].They are captured periodically by these devices installed on moving bodies as sequences of geographical coordinates and timestamps. Every day, enormous amounts of data are created and collected. Additionally, the movement of objects generally follows frequently repeated patterns. Trajectory pattern discovery has applications in movement prediction [2,3,4], region of interest discovery [5], and the study of traffic flow or congestion [6]. Therefore, extracting implicit and valuable patterns from vast databases of location data has attracted great interest in recent years.

This work focuses on extracting knowledge from vehicle trajectories through Sequential Pattern Mining (SPM). SPM is used to find sequences whose ratio of occurrences exceeds a user-defined minimum threshold [7,8,9]. The resulting frequently occurring sequences that are retrieved can be used to find connections between different objects or events. A similar concept can be extended to trajectory data, where sequential patterns are collections of geographical locations that several moving objects visit in a particular order. Therefore, applying SPM on trajectory data gives rise to frequently appearing sequences of locations, which provide insights into the travel pattern of the subjects. Analyzing and predicting travel behavior is facilitated by thoroughly understanding residents’ travel patterns, providing valuable insights into socioeconomic dynamics. These mining objectives are best achieved with taxi trajectory data since it is ofhigh quality, consistent, and has a wide distribution [10]. Hence, this work uses Microsoft T-drive taxi trajectory data [11,12].

Trajectories are essentially spatio-temporal records of vehicles. Performing SPM with vehicle trajectory is problematic for two reasons: (1) Spatial uncertainty and noisy trajectory recordings plague GPS technology. It is impossible to have vehicles visit the exact locations. For example, two vehicles may travel on the same stretch of road, but their recorded coordinates rarely match precisely. Therefore, finding frequent travel patterns necessitates some level of fuzziness. (2) SPM requires sequences of discrete items. However, each trajectory commonly contains hundreds and thousands of GPS points without any pre-defined segmentation, presenting a redundancy issue. Worst still, the trajectories recorded are far from discrete. Due to these characteristics of the trajectory data, many SPM methods now in use often result in enormous, repetitive, and indecipherable outputs [13].

Addressing these issues requires transforming trajectory data into formats compatible with current sequential pattern mining systems. An abstract method of segmenting the trajectories is essential, ensuring the fuzziness of data while reducing the size of the data and, at the same time, discretizing continuous data. Trajectory simplification methods such as grid-based or clustering-based segmentation can be applied to get discretized trajectories and remove spatial uncertainty, thereby preparing trajectory data for SPM algorithms [1,14,15]. Obtaining a smaller, less redundant set of sequential patterns can be resolved by enforcing constraints such as closed, maximal, or contiguous [7,16,17]. For example, by enforcing contiguous constraints, items in resultant patterns must also be adjacent in the original sequence. Such an approach can be observed in [18].

Until now, there has not been much comparative work that extensively examines and summarizes the performance of the various SPM types in the context of trajectory data. Furthermore, most of the recent works either focus on introducing trajectory data mining and provide little clues on the application of SPM on trajectory data, such as [1,19,20], or focus on the overview of SPM, such as in [7,21]. Therefore, through extensive experiments, this work presented the performances and behaviors of several SPM algorithms, namely GSP, CM-SPADE, PrefixSpan, CM-Clasp, CloFast, MaxSP, VMSP, and CM-SPAM. This work served as a reference for those interested in implementing the SPM algorithms in trajectory-related applications. The process of searching for and choosing a suitable algorithm is time-consuming. With such a reference guide, users can therefore pick the most appropriate algorithm based on the application scenario promptly.

The remainder of the paper is structured as follows: Section 2 introduces the definitions and problems of sequential pattern mining. Section 3 elaborates on representative sequential mining algorithms and constraint-based SPM based on their approaches, some of which were used in this comparative study. Section 4 describes the data processing procedure and experiment details. Section 5 presents and discusses the results of mining T-drive data using different SPM algorithms. Finally, this paper concludes with Section 6.

## 2. Preliminary and Problem Statement

This section clarifies some basic terminologies and definitions to better illustrate sequential pattern mining and its application on trajectory data.

### 2.1. Itemset, Sequences and Support

In a sequence database, sequential patterns refer to itemsets arranged according to their inherent orders, such as time of occurrence [22]. An itemset is a subset of items, whereas an item is an entity that can have properties such as date, time, size, colour, speed, coordinates, etc. Let $I=i_{1},i_{2},\ldots ,i_{n}$ be a non-empty set of items. An itemset $X=x_{1},x_{2},\ldots ,x_{m}$ is a set of items where X⊆I. A sequence s is an ordered list of itemsets denoted by $<X_{1},X_{2},\ldots X_{j}>$ where $X_{k}\subseteq I$ for 1≤k≤j and itemset $X_{k}$ is an element of sequence s. An item can appear more than once in different sequence elements, but only once in any element.

The length of sequences is the total number of items in all elements in a sequence s, denoted by length(s). The size of a sequence s is the total number of elements in s denoted by size(s). As an example, $s_{1}=<a,b,c>$ has $length(s_{1})=3$, or $|s_{1}|=3$ and $size(s_{1})=3$. $s_{1}$ can be described as a 3-sequence containing three items, whereas another sequence $s_{2}=<\left(ab\right),c>$ has a $length(s_{2})=3$ and a $size(s_{2})=2$. A sequence $s_{y}=<Y_{1},Y_{2},\ldots ,Y_{n}>$ is a subsequence of sequence $s_{x}=<X_{1},X_{2},\ldots ,X_{m}>$ and $s_{x}$ is also called a super-sequence of $s_{y}$ where $s_{x}$ contains $s_{y}$, i.e., $s_{y}\subseteq s_{x}$ if an only if there exists integers $1\leq j_{1}<j_{2}<\cdots <j_{n}\leq m$ such that $Y_{1}\subseteq X_{(}j_{1}),Y_{2}\subseteq X_{(}j_{2}),\ldots ,Y_{n}\subseteq X_{(}j_{m})$. For instance, $s_{2}=<a,b,c>$ is a subsequence of $s_{3}=<a,b,c,d>$ and $s_{3}$ is said to be a super sequence of $s_{2}$.

A sequence database SDB is a set of tuples <id,s> where id is the identifier of sequences and *s* is a sequence. An example of a sequence database containing four sequences is shown in Table 1. Taking this idea further, it can be observed that each id represents a specific vehicle, and each sequence represents a vehicle’s collective trajectory over time.

### 2.2. Motivation

The problem of frequent itemset mining to discover frequently appearing items in a relational database was proposed by Agrawal, Imieliński, and Swami in 1993 [23]. Along with the concept, the Apriori algorithm was proposed. Sequential pattern mining extended from itemset mining and was introduced by Agrawal and Srikant in 1995 [9]. It considered orders by discovering subsequences in a set of sequences and aims to discover frequent sequential patterns. A sequential pattern is defined as a set of itemsets arranged in a sequence database occurring sequentially in a specific order, i.e., time. The number of tuples or the number of ids in *D* is denoted as |D|. Support is the frequency of a subsequence $s_{a}$ in *D* is denoted by sup denoted by (1):

(1) $$sup=\frac{\#\;of\;sequences\;contains\;s_{a}}{Total\;\#\;of\;sequences\;in\;D}$$

Minimum support threshold min_sup is an user-defined parameter. The result of frequent pattern mining requires the support of subsequences to meet the user-defined minimum threshold min_sup for a subsequence to be considered *frequent*. The task of frequent itemsets mining in a large transaction database is challenging as a massive number of distinct single items can give rise to many combinations. Consider a pattern of length *m*. Such pattern gives m1 sub-patterns of length 1; and m2 sub-patterns of length 2 and so on until mm sub-patterns of length *m*. Therefore, the total number of sub-patterns in a pattern of length *m* will be m1+m2+⋯+mm=2m−1. Suppose C denotes the power-set of a set of items in *I*. As shown by the (2), the goal of mining is to discover recurring itemsets among $|C_{min\_sup}|$ different possibilities:

(2) $$|C_{min\_sum}|=\sum_{k=1}^{m}\frac{m}{k}-1=2^{m}-1$$

Hence, if $S_{n}$ is the possible frequent sequences within *n* itemsets, then the value of $S_{n}$ is represented by (3):

(3) $$S_{n}={\left|C_{min\_sup}\right|}^{n}={\left(2^{m}-1\right)}^{n}$$

Apriori exhibits a downward closure property, in which the key concept is that the support of a k-itemset is less than a threshold t if the support of all its subsets is less than that t. In other words, all subsets of any frequent itemset must also be frequent [22]. As a result, all of an itemset’s extensions will also be rare if the itemset itself is rare in the database. The foundation for frequent pattern mining algorithms, such as sequential pattern mining, is provided by this characteristic.

### 2.3. Trajectory Data

Trajectory data is a type of spatio-temporal data that describes the paths traveled by moving objects in space over time [1,24] One example of such data would be the route taken by a taxi from one point, i.e., a pick-up point, to another point, i.e., a drop-off point. Trajectory data is defined as a set $T=\left(x_{1},y_{1},t_{1}\right),\left(x_{2},y_{2},t_{2}\right)\ldots \left(x_{n},y_{n},t_{n}\right)$ of trajectories such that $t_{i}<t_{\left(i+1\right)}$ for all i∈1,…,n and each ($x_{i},y_{i},t_{i})$ is a data point denoting the geo-spatial coordinates of a moving object in the form of latitude ($x_{i}$) and longitude ($y_{i}$) at time $t_{i}$. A sub-trajectory *t* is a subset of *T* such that *t* contains all data points in *T* between the time interval $\left[t_{i},t_{j}\right]$ where $t_{i}<t_{j}$. Trajectory data must be organized in a structured sequence database to extract frequent trajectory patterns. A sequence database SDB is a collection of tuples <id,s> where each id identify a sequence *s*. Here, the id identifies the trajectory data of each vehicle, and s is the corresponding trajectories $T=\left(x_{1},y_{1},t_{1}\right),\left(x_{2},y_{2},t_{2}\right)\ldots \left(x_{n},y_{n},t_{n}\right)$. Geographical coordinates and timestamps can be interpreted as a time-ordered array of itemsets. Considering a vehicle can only be at a location at once, a trajectory, unlike a standard sequence database, cannot appear numerous times in different itemset in a sequence. Therefore, a trajectory database must contain sequences with itemsets arranged in ascending order based on the timestamp. In conclusion, the purpose of sequential pattern mining of trajectory data is to find common sequences of locations that different moving objects visited in ascending order of timestamps.

### 2.4. Trajectory Segmentation

SPM works with sequences of discrete items. There is no pre-defined segmentation of trajectories, and the trajectories recorded are far from discrete. Consequently, many existing SPM approaches generate redundant and incomprehensible patterns. This complex nature of trajectory data requires a segmentation method that splits them into disjoint, smaller, and discrete sub-trajectories before any sequential pattern extraction can proceed. Vehicles may visit locations that do not exactly match GPS coordinates but are close geographically and can be considered part of the same pattern [24]. Extending from that, the trajectory of the typical sequential pattern will follow a similar spatial path but will not necessarily be the same. There should therefore be some fuzziness in frequent patterns. Therefore, frequent patterns should have a certain level of fuzziness.

Methods such as the grid-based [19,25,26] or clustering-based method [27,28] may be employed to segment the trajectories in spatial aspects in preparation for further studies. For example, a work by Tsoukatos and Gunopulos in 2001 used the grid-based method, where ordered sequences of rectangular regions are used to define sequential patterns [25]. This method generally groups the GPS points nearby using grids. The entire city, or any space containing all the trajectory data, is divided into grids with user-defined grid sizes. Each grid is considered a sub-region, each with its unique ID. GPS points within this sub-region are denoted by the same grid ID, thereby transforming trajectories from a series of points to a series of grid IDs. Then, from these sequences of grids, frequent sequential patterns are found.

Contrarily, clustering-based methods are unsupervised methods that cluster sub-trajectories based on metrics such as time intervals or distances depending on the clustering algorithm. After clustering, points belonging to the same cluster will be labeled with the cluster ID, similar to the grid-based method. Eventually, trajectories will be transformed into a series of cluster IDs. On the other hand, clustering methods rely on the nature of the data to determine parameters such as the number of centroids *k* (for *k*-means clustering) or *minPts* and *epsilon* (for density-based clustering methods like DBSCAN and OPTICS). Prior work by Karsoum et al. compared these two approaches [15]. Results demonstrate that although the density-based method has a faster execution time, grid-based methods can discover more hidden patterns with the same parameters (i.e., min_sup).

## 3. Sequential Pattern Mining Algorithms

Pattern mining algorithms differ in several ways: (1) the candidate search method, such as breadth-first or depth-first search, and (2) the database representation. For example, the database described previously is an implementation of a horizontal database. Sequential pattern mining is computationally more intensive than itemset mining as many intermediate candidates or subsequences must be generated and verified during the process. A typical sequential pattern mining algorithm always aims to find all the patterns given a database and the min_sup threshold. When mining long sequences, having massive databases, or small min_sup, the computational resources required may become the limiting factor. According to (2), a frequent 100-sequence will give rise to $2^{100}-1$ frequent subsequences with a min_sup of 1. Also, the algorithms sometimes demand too much computing power, preventing them from completing the search. Many efforts have been made to increase the efficiency of algorithms. Some directions of optimization can be: (1) reducing intermediate candidate size, (2) fewer database scans, (3) limiting the search space, and (4) optimizing candidate generation and support counting phase.

Besides computational inefficiency, the algorithms often output a massively large number of frequent patterns. Redundant and meaningless patterns in the output caused users to spend additional time and effort searching for patterns of interest. Mining trajectory sequential patterns, which often have long sequences, poses the challenge of obtaining a smaller, less redundant set of sequential patterns. These challenges can be overcome by using constraint-based SPM. This diversion of SPM allows results to be summarized as a concise representation of a complete set of frequent patterns without extracting the entire set meaningfully.

This section first introduced three main types of search techniques and their representative algorithms, followed by three different constraints and the algorithms that enforced them.

### 3.1. Breadth-First Search

The Apriori algorithm was proposed to deal with the problem of frequent itemset mining [23]. The algorithm is designed for mining frequently occurring itemsets by applying the downward closure property, which states that if an itemset is infrequent in the database, all its extensions would also be infrequent [22]. It is particularly useful during pruning by greatly reducing the search space. However, Apriori does not account for orders, causing it to fail in situations where orders matter, such as time-series data and text. Sequential pattern mining was subsequently introduced [9]. Prior to expanding them to longer sequences, AprioriAll first determines which item is frequently observed in the database. It proceeds in a two-step manner: candidate generation followed by support counting. In candidate generation, the algorithm first searches for the frequent 1-sequences (i.e., sequences containing a single item) and generates 2-sequences by extending the 1-sequences.

Similarly, 3-sequences are generated with 2-sequences. The process continues until no further extensions can be made. This approach is also known as the level-wise or breadth-first approach [7,22]. Each n-sequence extension scans the whole database. The search space with such approaches can become monumental as they always consider the worst-case and explore all possible sequences [7]. In the support counting phase, AprioriAll employs a hash tree to count the generated sequential patterns and remove unwanted patterns. Various improvements have later been proposed to increase the efficiency of the Apriori-based algorithm, but techniques such as the two-step approach and hash-tree-based support counting in AprioriAll remain inspirational for newer algorithms.

The authors of the Apriori algorithm, Agrawal and Srikant, proposed an improved version named Generalized Sequential Patterns (GSP) in 1996 [29]. GSP adopts a similar type of horizontal database as Apriori and also uses the breadth-first search for frequent sequential pattern discovery. GSP attempts to generalize sequential pattern mining by employing time restrictions, sliding time windows, and taxonomies in sequential patterns [22]. Like AprioriAll, GSP follows a two-step approach where all candidates are generated prior to support counting. However, GSP keeps all k-sequences in memory to extend and generate k + 1-sequences [7]. By merging smaller patterns, this tactic allows GSP to generate candidates without repeatedly visiting the database. Although this reduces the number of database scans, much time and space is wasted on non-existent candidates. In the second step, GSP performs multiple database scans to calculate the support, which is very costly for large databases.

### 3.2. Depth-First Search

Unlike breadth-first search, the depth-first search algorithm starts with sequences containing single items, i.e., 1-sequence, and recursively extends one of the sequences until exhausted. Then, the algorithm returns and extends another 1-sequence to generate other sequential patterns. This search strategy was employed in the SPADE (Sequential Pattern Discovery Algorithm Using Equivalence Classes) by Zaki in 2001 [30]. SPADE creates an IDList for each item, indicating that particular item’s position in the respective sequence. An IDList is a vertical database representation. All IDLists are generated together by scanning the horizontal database once. The horizontal database can be reconstructed from corresponding vertical databases. The support counting step is greatly simplified as all frequent patterns can be enumerated by performing the joins of IDLists, hence calculating the support of any pattern directly. Subsequently, it checks the cardinality of the resulting id-list against min_sup to ensure that all subsequences of the resulting sequence are frequent. Adopting such approaches improved the performance significantly compared to breadth-first search algorithms without the need to perform multiple databases or maintain candidates in memory.

However, the issue of the non-existence candidates persisted as the candidate generation procedure made no references to original databases. Furthermore, when a pattern appears in many sequences, the join operation of IDList remains costly in a large database, especially a dense one or long sequence database. Co-occurrence pruning was introduced by Fournier-Viger et al. in CM-SPADE to minimize join operations [31]. A co-occurrence map was created during the initial database scanning, which stores all frequent 2-sequences. If the last two items of any sequence are not in the co-occurrence map, the sequence is eliminated directly without constructing IDLists, thus reducing the number of join operations.

Another strategy using bitmap representation of vertical databases is employed in SPAM [32]. For each item, a bitmap with a bit of 0 or 1, depending on the appearance of such an item in each pattern, is generated. Such a data layout makes SPAM perform efficient support counting and is capable of online outputting the resultant pattern. However, SPAM is relatively space-inefficient. It needs to fit the entire database and data structure into memory. This makes large trajectory data possibly incomprehensible when memory resources are limited. Similarly, co-occurrence pruning was added in CM-SPAM to enhance the performance of SPAM.

### 3.3. Pattern-Growth

Besides utilizing a vertical database, another way of optimizing the frequent pattern mining process is by establishing a frequent pattern tree (FP-tree) structure projected database, which is a more efficient variation of the horizontal database and extends from the prefix tree during the depth-first search [33,34]. Such an approach eliminates the costly task of candidate generation as it gradually grows trees of frequent itemsets during projected database generation. The original database is divided into a series of projected databases, with each itemset projected to no more than one of the projected databases. The size of the resulting database is always less than that of the original database. Next, this method starts from a frequent pattern of length 1, or suffix pattern, and recursively extends the pattern from sub-databases consisting of the set of frequent items co-occurring with the suffix pattern [33]. Larger patterns form as the algorithm recursively concatenates items to suffix patterns, eventually resulting in a constructed FP-tree. During the process, sets of frequent patterns under the same suffix pattern are ordered in descending supports. The FP-tree-based mining approach achieves efficiency in three ways: (1) Using a highly condensed database that reduces the number of database scans to two, hence avoiding multiple database scans; (2) Using a pattern fragment growth method to avoid massive candidate sets; and (3) Using a partitioning-based, divide-and-conquer method to reduce colossal search space.

However, the FP-tree is unsuitable for sequential mining as sub-sequences with different orderings cannot be re-ordered and considered the same pattern, leading to a vast, non-collapsible database. In the worst-case scenario, database projection will require copying almost the entire database. Based on the idea of FP-tree, Pei, Han et al. developed PrefixSpan (Prefix-projected Sequential pattern mining) and extended it to sequential pattern mining [35]. In PrefixSpan, the corresponding postfix subsequences of prefix subsequences in sequence databases are recursively projected into smaller databases. Upon completion, each projected database considers only frequent local patterns to grow the sequential pattern trees. When the database is huge, the main-memory-based pseudo-projection techniques in PrefixSpan, which consider the projected database as a set of pointers on the original database, can reduce the computational cost for projection.

### 3.4. Closed Constraints

Closed frequent patterns represent the largest sub-sequences common to sets of sequences [7]. Such patterns are lossless, which means the whole set of sequential patterns can be reconstructed from the resulting patterns. Given a dataset *D*, a pattern *X* is a closed frequent pattern if *X* is frequent and there exists no proper super-pattern *Y* such that *Y* has the same support as *X* in *D*, i.e., sup(X)>sup(Y) for all Y⊃X. The idea of a closed pattern was first introduced by Pasquier et al. in 1999, together with the frequent closed itemset discovery algorithm AprioriClose (or A-Close) [36]. Subsequently, Yan et al. proposed CloSpan (Closed Sequential Pattern Mining), which targets closed sequential patterns [37]. CloSpan was extended from PrefixSpan and adopted a two-step approach. The first step generates a candidate set based on the concept of equivalence of the projected database, followed by pruning all non-closed sequences in the second step. A hash-based technique for pruning where the hash function is the support of a sequence. CloSpan outperforms PrefixSpan and can mine long sequences in a large database even with low min_sup where PrefixSpan failed. ClaSP (Closed Sequential Patterns algorithm) was then proposed by Gomariz and Campos et al. [38]. It marries the idea of vertical databases from SPADE and the heuristic pruning approach from CloSpan. However, ClaSP needs to maintain previous candidates in the memory for sequence pruning, which is rather memory-intensive. Similar to CM-SPADE, Co-occurrence map was also implemented in CM-ClaSP by Fournier-Viger et al. [31]. Unlike the aforementioned approaches, CloFAST combines the idea of *sparse id-list* and *vertical id-list* to enable rapid counting of sequential patterns [39]. The properties of such combinations improve the memory-intensive situation in ClaSP by allowing a one-step approach for both sequence closure checking and search space pruning. Specifically, sparse id-list is used for closed frequent itemset mining, and vertical id-list is used for closed sequence pattern generation.

### 3.5. Maximal Constraints

For dense databases or databases with long sequences, closed sequences may still produce a large set of patterns. Therefore, maximal patterns are introduced to address such issues further. Maximal patterns are sets of sequential patterns that do not appear in other sequential patterns. By definition, a pattern *X* is a maximal frequent pattern in dataset *D* if *X* is frequent in *D*, and there exists no sequence *Y* where X≠Y, which is a super-sequence of *X*. A set of maximal sequential patterns is always smaller than the set of closed sequential patterns and all sequential patterns, i.e., MS⊆CS⊆FS. Unlike closed patterns, maximal patterns are not lossless. Similarly, different strategies are employed for finding maximal sequential patterns or itemsets. For example, AprioriAdjust and FMMSP [40] uses breadth-first search algorithms, MaxSP [41] and DIMASP [42] use pattern-growth algorithms, and VMSP [43] uses depth-first algorithms with a vertical database. MaxSP adopted PrefixSpan’s projected database mechanism. To tackle the memory inefficiency of previous algorithms, MaxSP determines whether a frequent pattern is maximal without retaining previously found patterns in the memory. It incorporates a checking mechanism consisting of verifying maximal-backward-extensions and maximal-forward-extensions [41]. With these, MaxSP can output results directly. A more efficient method, VMSP, uses vertical databases instead of costly database projection generation in MaxSP. VMSP relies on three approaches: exclusion of non-maximal patterns, validation of forward maximal, and co-occurrence map candidate pruning to achieve its efficiency [43].

### 3.6. Contiguous Constraint

Contiguous constraint requires items in resultant patterns must also be adjacent to each other in the original sequence. Two sequences *Y* = <$y_{1},y_{2},\ldots ,y_{i}$> and *X* = <$x_{1},x_{2},\ldots ,x_{i}$> where *X* is a contiguous subsequence of *Y* denoted by X⊑Y if and only if there exist integers $k_{1},k_{2},\ldots ,k_{i}$ where $1\leq k_{1}<k_{2}<\ldots k_{i}\leq \;j$, and $x_{1}=y_{k_{1}},x_{2}=y_{k_{2}},\ldots ,x_{i}=y_{k_{i}}$. It also implies that *Y* is a super-sequence of *X*. GSP was one of the first few algorithms to incorporate the idea of gap constraints and contiguity in sequential patterns [29]. The constraint is well-suited to a particular trajectory data mining goal, assuring that the resulting trajectory patterns will always follow the actual trajectory. Without this constraint, resultant elements in sequential patterns, which represent the locations in a trajectory, can jump from one position to another. Some algorithms, such as VMSP and CM-SPAM, inherently allow specifying the number of gaps between itemsets, with no gaps allowed, suggesting a contiguous constraint.

## 4. Methodology

This section focuses on the design of the experiments to evaluate the performances of the algorithms discussed in the previous section. Several representative algorithms will be chosen to participate in the experiment using a large trajectory dataset, T-drive data. Algorithms are chosen based on their search heuristics, database implementations, and constraints applied to study how these affect the mining result.

All experiments were performed on a machine with a Linux system, running on an Intel(R) 6-Core (TM) i7 CPU processor at 3.20GHz, 16GB DDR4 memory with low latency Solid State Drive (SSD) as storage. Data cleaning and processing were done with Python version 3.10.2. SPMF (Sequential Pattern Mining Framework) (version 2.52) was used to run the algorithms. SPMF is a Java-based open-source data-mining library running on 64-bit Java Running Environment (JRE) which has been deployed in many researches [44]. This library ensures maximum comparability and reproducibility of the experimental results. However, the performances may vary considering the default mechanism of JAVA, specifically the controlled memory allocation and the garbage collection mechanisms. Also, it is assumed that the algorithms in SPMF are correctly implemented and optimized.

### 4.1. Datasets and Pre-Processing

**Figure 1:**
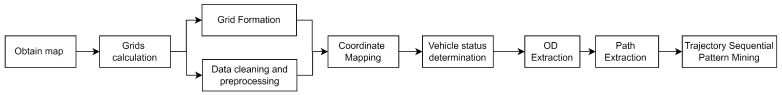
Procedure of generating discrete trajectory database for sequential pattern mining.

This study will use the Microsoft T-drive data, which contains the raw GPS trajectories of 10,357 taxis in Beijing, China, between 2 February 2008 and 8 February 2008 [11,12]. There are 15 million GPS recorded trajectories, adding up to 9 million kilometers. The dataset is a good representation of typical real-life trajectory records. Table 2 below gives an example of the trajectory data. Figure 1 briefly overviews the whole data processing procedure to prepare trajectory data for subsequent sequential pattern mining. The process started with obtaining the map data of the study area. The study area needed to be divided into grids with user-defined grid sizes for trajectory segmentation. Based on the size of one grid, it was possible to calculate the total number of grids required to encompass the study area. With the size of the grid and the total number of grids, grid formation was performed to calculate the geographical coordinates of the center of grids such that the grid center coordinates can represent any GPS point within the grid. At the same time, raw trajectory data must be cleaned and processed to remove unwanted trajectories. Detailed procedures for data cleaning and preprocessing were elaborated in the following sub-sections. When trajectories and grids were ready, it was possible to map all GPS points within the trajectory to grids to represent GPS sequences coordinated with a series of grids. The next step would be determining the status of the taxi considering the events such as parking, shift change, and traffic congestion. In these scenarios, vehicles would be in a stationary position. This step is crucial for determining a trajectory’s origin-destination (OD extraction) and subsequent path extraction, as data was recorded continuously throughout the period. The above steps prepared the data for subsequent sequential pattern mining.

### 4.2. Grid Formation

The map data for Beijing, China, is obtained from OpenStreetMap. Grid-based trajectory segmentation is performed with Python version 3.10.2 on Microsoft VSCode. This study uses a grid size of 500 m × 500 m. Based on [15], which investigated the impact of grid cells on the result, a larger grid size would reduce the execution time since the number of grids is reduced. The choice of grid size should consider factors such as the characteristics of trajectory data (i.e., length and spread of trajectories) and study area size. A larger grid size results in more points belonging to the same cell, and hence, fewer patterns are found by pattern mining algorithms. Specifically, one point may belong to the *i*th grid, and the other may belong to the (i+1)th grid, but with a large grid size, all these points belong to the same grid. As the total number of grids decreases, the resultant sequential pattern found can be too generalized to be useful. For instance, a grid size of 1 km × 1 km was considered large for the T-drive dataset. Nevertheless, when the grid size is too small, in the worst case, most grids contain at most one data point, which defeats the purpose of trajectory segmentation. For this dataset, the grid size of 250 m × 250 m is too small, and most algorithms fail to give valid results even at higher min_sup. Eventually, this study chose 500 m × 500 m as the grid size.

The maximum and minimum geographical coordinates of the study area, Beijing city, are (113.75194, 22.447837) and (114.624187, 22.864748), respectively. These points form a rectangular region divided equally into 32,984 grids. The next step is assigning an ID to each grid and calculating the geographical coordinates of the respective grid centers. Considering Earth’s spherical shape, changes in the latitude and longitude of each grid center are not constant as on a flat plane. Given the average radius of Earth is 6,371,004 m, let R represent the radius, *x* be the grid size defined in meters, and lat_1 and $lat_{2}$ be the minimum and maximum latitudes of the study area; then, (4) and (5) can be used to calculate the changes in latitude and longitude. All recorded taxi coordinates are mapped to each grid. Here, we have the original coordinates, the grid ID it belongs to, and the coordinates of the grid center.

(4) $$\Delta Lon=\frac{360x}{2\pi Rcos(\frac{\pi (lat_{1}-lat_{2})}{360})}$$

(5) $$\Delta Lat=\frac{360x}{2\pi R}$$

### 4.3. OD Extraction

T-drive data consists of the ID of each taxi, the DateTime, and the longitude and latitude of locations. The geographical locations of each taxi are continuously recorded. For each taxi, each record’s DateTime and geographical location are compared with the next. Hence, we are then able to label the status of each record as moving or stationary. If the next time point coordinate remain the same as the current time point, the taxi is not moving. If the status at time t−1 equals the status at time t+1 and the status at time *t* is not equal to any, the record at *t* is considered abnormal. The status of the record at *t* is then corrected to that of t−1.

**Figure 2:**
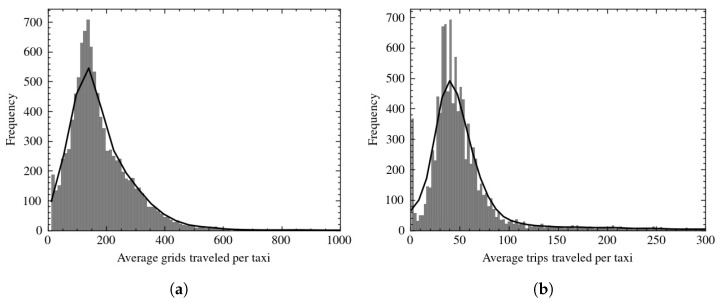
Distribution of data after segmentation. (**a**) Average grids traveled per taxi. (**b**) Average trips traveled per taxi.

Taxis may be running, parking, or waiting for traffic. Here, it must be noted that when the taxi is in a prolonged stationary state suggests a change of shift or is in offline status. In this case, only trajectories between two parking events are wanted, and time spent waiting for traffic should be distinguished from parking events. The first step is to identify moving events between two parking events. Based on the pre-processed data, where the status of each record is marked as 1 or 0, if a taxi remains in the same grid for 10 min or more, it is considered as parking and marked 0. Finally, each taxi’s origin-destination (OD) data for each trip is extracted. Each record, instead of the taxi’s location at each timestamp, had become a single trip made by the taxi. There are at least 10 minutes of stationary phase between each trip. Each row consists of the Taxi ID, start time and end time of the trip, the grid ID of origin, and a list of locations in the form of grid ID involved in the trip. The final dataset contained 592,982 trips traveled by 10,226 vehicles. Figure 2 shows the frequency distribution of grids and trips traveled per taxi, respectively. Overall, taxis traveled an average of 187.66 grids and 57.98 trips during data collection.

## 5. Experimental Results and Analysis

This section presents the results and analysis of comprehensive experiments. This paper intends to evaluate each algorithm based on the following properties: runtime, memory usage, and the number of patterns generated. Each algorithm’s runtime was measured from the time the algorithm started to the time it returned a result. Memory usage was obtained with the Java class Runtime. Each algorithm was repeated with the same parameters five times, with the results averaged to obtain the final runtime and memory consumption. The number of patterns generated depends on the min_sup parameters. Hence, each algorithm would be run with min_sup values of 0.1, 0.2, 0.3, 0.4, 0.5, 0.6, and 0.7. VMSP and CM-SPAM can be run with and without gaps allowed between candidate sequences. When no gap is allowed, it forces the resultant pattern to be consecutive, i.e., a contiguous constraint. Experiments are conducted with bash scripts running in a Linux environment with breaks between each run and consider sufficient JVM warm-ups beforehand.

The data for runtime (s), RAM consumption (Mb), and the number of output patterns are shown in Table 3, Table 4 and Table 5, respectively. All algorithms give no pattern output at min_sup of 0.7, suggesting that no grid has 70% of all trajectories passing through. Among all, MaxSP failed at min_sup is at 0.2 or less. Only CM-SPADE and algorithms with contiguous constraints enforced can mine at a min_sup of 0.1. The considerable number of patterns mined by CM-SPADE indicated that non-constrained algorithms might struggle with either time, memory, or storage space, given the massive number of outputs.

Algorithms that failed experienced either Java heap space problems, extremely long run times, or exceptionally high RAM usage if min_sup is too low, i.e., below 0.2. In these scenarios, algorithms either threw an execution exception, were killed when runtime exceeded 3 h, or when output occupied more than 300 GB of storage space. These algorithms were marked with “Failed” in the result tables.

The number of outputs presented a challenging task for processing and interpretation. Theoretically, max and closed constraints should return a concise representation of the output, yet algorithms returned a similar number of outputs with the T-drive dataset. This observation revealed that the characteristics of the dataset determined these constraints’ effects. Trajectory data might be too distinct to be compressed compared to other forms of data.

Between three algorithms without constraints: GSP, PrefixSpan, and CM-SPADE, GSP observed a sharp increase in runtime as min_sup decreased to 0.1, while PrefixSpan and CM-SPADE remained relatively stable. However, CM-SPADE consumed more RAM but could mine sequences at min_sup of 0.1, where PrefixSpan failed. PrefixSpan utilizes main-memory-based pseudo-projection techniques, which reduce RAM consumption significantly, as demonstrated by experiment results. PrefixSpan trades execution time for less RAM consumption. PrefixSpan might be able to finish execution if more time were given. On the other hand, CM-SPADE had a shorter, more stable execution time at the expense of higher RAM consumption time. The choice between PrefixSpan and CM-SPADE would depend on the computational resources available.

As closed-constraint algorithms, CM-ClaSP and CloFast were similar in terms of RAM usage. However, CloFast was significantly faster. The number of closed sequential patterns discovered differs slightly between the two algorithms, which is attributed to different strategies employed. Strangely, the number of closed patterns found by CloFast was greater than those without constraints. Experimental figures further suggest that the closed pattern did not work well with trajectory data.

Conversely, maximal SPM algorithms like MaxSP and VMSP adopt very different candidate generation and pruning approaches. MaxSP can generate output directly without in-memory candidate maintenance, whereas VMSP opts for the common candidate-maintain-and-test approach. The approaches generated a notably different number of patterns. VMSP mined more maximal sequential patterns with less RAM consumption and shorter execution time, while MaxSP has the highest RAM consumption and longest execution time among all algorithms. VMSP, therefore, is more efficient considering the size and sequence length of typical trajectory databases.

Contiguous constraints were performed with VMSP and CM-SPAM. Their respective gap constraints were set to no gaps allowed. Their runtime differed by a few seconds, while VMSP had consumed lesser RAM than CM-SPAM.

**Figure 3:**
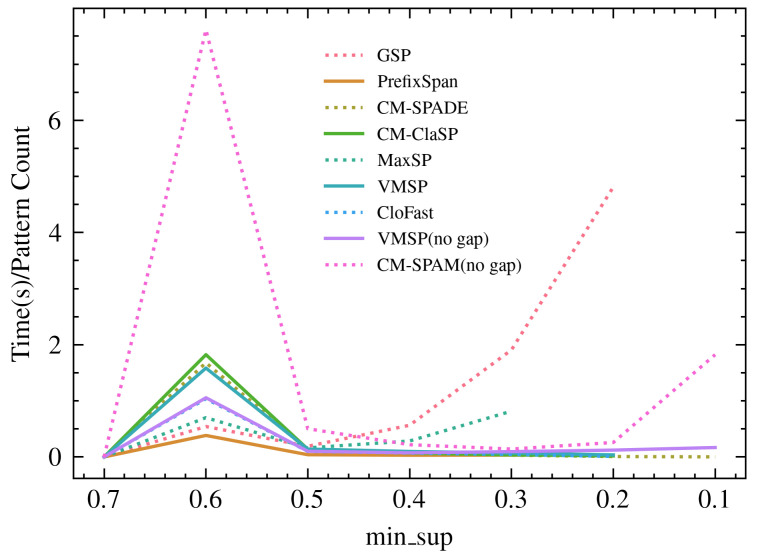
Comparison of algorithm efficiency through time(s) over pattern outputs at different min_sup.

However, judging the algorithm’s efficiency simply through runtime or the number of pattern outputs was unfair. Figure 3 depicts algorithm efficiencies using runtime per pattern. When compared to algorithms that found a similar number of patterns (i.e., VMSP (no gap) and CM-SPAM (no gap)), GSP performed better at most min_sups, particularly at 0.6, 0.3, and 0.2. As a closed-constrained algorithm, CM-ClaSP was the second most efficient algorithm besides GSP at a min_sup of 0.6, while MaxSP became the second at a lower min_sup of 0.3. CM-ClaSP was the best-performing among algorithms without constraints. However, MaxSP and GSP failed to complete the task as min_sup was further reduced. Therefore, at low min_sup, CM-SPAM (no gap) became the most efficient algorithm.

**Figure 4:**
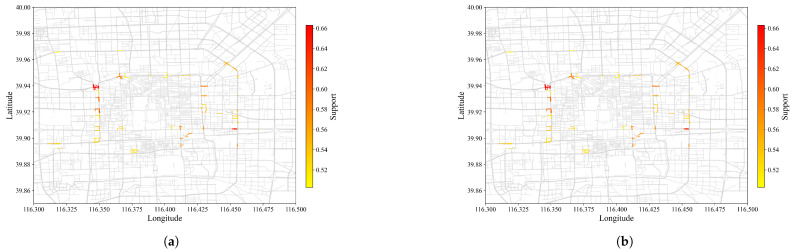
Output of SPM (min_sup = 0.5) against Beijing road map. (**a**) Without constraint (**b**) With constraint. Color changes from yellow to red as support increases. Higher support suggests more vehicles trajectories overlapping at the particular road segment.

**Figure 5:**
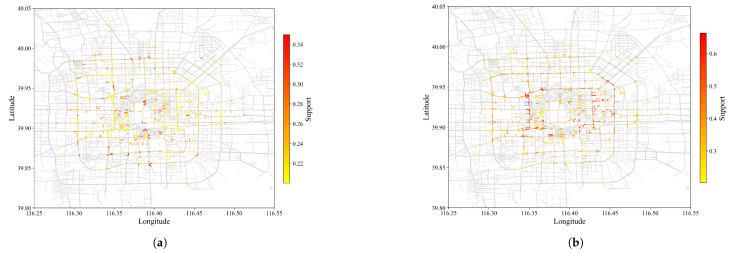
Output of SPM (min_sup = 0.2) against Beijing road map. (**a**) Without constraint (**b**) With constraint. Color changes from yellow to red as support increases. Higher support suggests more vehicles trajectories overlapping at the particular road segment.

**Figure 6:**
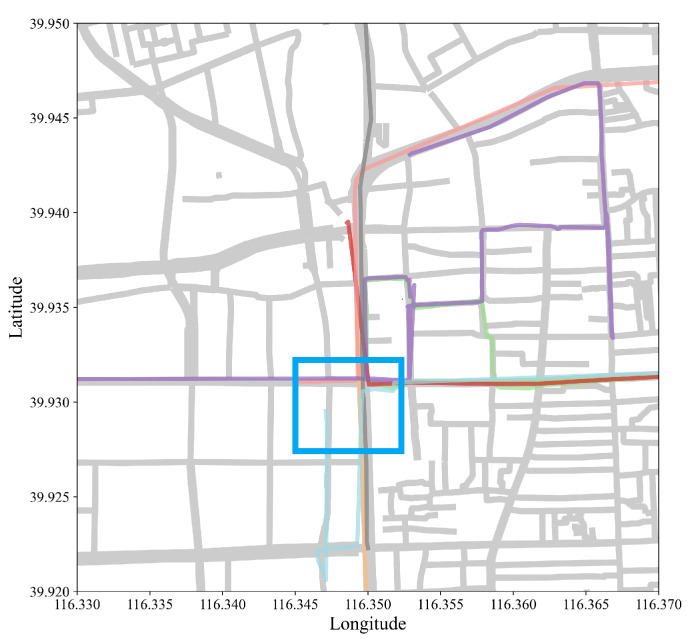
An example of sequential pattern mined in the blue boxed region (Figure 5b) with actual trajectories(colored lines) against the roads (grey lines).

Figure 4 and Figure 5 visually compared SPM results at min_sup of 0.2 and 0.5, with and without contiguous constraints plotted against actual roads of Beijing. Results from CM-SPADE and CM-SPAM were used, respectively. At min_sup of 0.2, the outputs of three algorithm groups, non-constraint based, closed constraints, and max constraints, fall in the range of 7628 to 8068, except for GSP, which produces a very concise result similar to that of contiguous constraint algorithms. Contiguous constraints give an exceptionally low output count, which makes the comparison meaningful. Higher support denoted that more vehicle trajectories traveled on the road segment. A red-colored road segment suggested a large volume of vehicles. CM-SPADE was selected for visualizations, representing the results of algorithms producing outputs within the 7628 to 8068 rang. Results were distributed within the 3rd Ring Road, which is a city ring road that encircles the center of Beijing. The solid red box outlined in blue in Figure 5b is one of the sequential patterns mined. The road segment in the blue box is the junction between the West Second Ring, which is notorious for traffic congestion in Beijing, and Chegongzhuang Road. Figure 6 exemplified the SPM result by plotting some trajectories that supported the pattern boxed out. The same road segments were found in these trajectories. Trajectory pattern mining tries to mine for repeated patterns, such as frequently traveled road segments. More overlapping trajectories give more support.

The high-support regions colored red and captured by SPM with and without constraints were highly similar at min_sup of 0.5. While with min_sup of 0.2, SPM with constraints captured more high-support road sections as contiguous constraints gave a more concise representation of results. The resultant pattern discovered by SPM without contiguous constraints tends to be duplicated. When the contiguous constraint was enforced, a single pattern could represent multiple patterns.

## 6. Conclusions

The continuity and uncertain nature of trajectory data make it distinctively different from typical transactional data, which requires additional trajectory segmentation procedures to allow applications of SPM for various purposes. This work outlined the method of dealing with trajectory data of such a nature. Subsequently, several representative algorithms were chosen to participate, including GSP, PrefixSpan, CM-SPADE, CM-SPAM, CM-CLasp, CloFast, VMSP, and MaxSP, in the sequential pattern mining comparative experiment using a large trajectory dataset, the Microsoft T-drive trajectory data. Algorithms are selected based on their search heuristics, database implementations, and constraints. Comparative experiments are performed to understand how different implementations and parameters affect SPM results. The runtime, RAM consumption, and the number of pattern outputs were collected. The results were further visualized on the actual map, which uses traffic congestion as an analytic example. These experimental results demonstrate that contiguous constraints are more relevant in the context of trajectory data than closed and maximal constraints, as contiguous constraints can provide a concise representation similar to constraint-less SPM and allow algorithms to perform well under extremely low min_sup when other algorithms fail to. Among all non-constraint-based algorithms, CM-SPADE shows decent performance at low min_sup and stable control of runtime and memory consumption with varied parameters. PrefixSpan is a suitable choice when RAM resources are limited, but there is plenty of storage space. This work performed sequential pattern mining using taxi trajectory data and visualized the result of SPM on the actual road in Beijing, China. The results highlighted road segments frequently traveled by taxi, which implies congested regions. Results from SPM can be further extended to other forms of analysis, such as next-location prediction. Employing trajectory map-matching techniques can further improve the accuracy of pattern mining. This study can be used as a guide for academics and professionals when determining the most suitable SPM algorithm for applications that involve trajectory data. Future experiments can investigate patterns’ quality and dive deep into reasons for inconsistency in pattern output between algorithms using similar constraints or parameter settings. A comparative study of other specialized forms of pattern mining, such as time constraints and top-k, can be considered.

## Figures and Tables

**Table 1:** An example of sequence database.

*id*	Sequence
1	<a,b,c>
2	<(ab),c>
3	<(ab),c,d>

**Table 2:** A snippet of original Microsoft T-drive dataset.

Taxi Id	Date Time	Longitude	Latitude
1	2008-02-02 15:36:08	116.51172	39.92123
1	2008-02-02 15:46:08	116.51135	39.93883
⋯	⋯	⋯	⋯
10,357	2008-02-08 17:26:51	116.72877	40.01143

**Table 3:** Runtime (s) of Algorithms with different minimum support (min_sup) categorized by their constraints.

Constraint	Algorithm	*min_sup*						
		0.7	0.6	0.5	0.4	0.3	0.2	0.1
No	GSP	1.66	1.62	8.76	67.73	581.82	2986.78	Failed
No	PrefixSpan	1.17	1.15	1.81	3.69	19.78	272.52	Failed
No	CM-SPADE	5.39	5.04	5.72	7.58	18.74	52.93	436.35
Closed	CM-ClaSP	5.81	5.47	6.34	8.50	22.03	143.45	Failed
Closed	CloFAST	24.45	22.84	23.10	26.16	42.85	162.80	Failed
Max	MaxSP	1.97	2.10	7.26	28.11	310.01	Failed	Failed
Max	VMSP	4.73	4.75	6.43	11.67	38.38	275.12	Failed
Contiguous	VMSP(no gap)	3.11	3.11	4.55	8.30	27.45	71.933	203.05
Contiguous	CM-SPAM(no gap)	3.41	3.16	4.65	8.74	28.78	75.26	210.26

**Table 4:** RAM consumption(MB) of Algorithms with different minimum support (min_sup) categorized by their constraints.

Constraint	Algorithm	*min_sup*						
		0.7	0.6	0.5	0.4	0.3	0.2	0.1
No	GSP	242.95	238.47	378.50	395.95	582.18	1197.63	Failed
No	PrefixSpan	147.98	178.37	374.3	365.03	430.45	596.19	Failed
No	CM-SPADE	2476.88	2465.66	1885.88	1522.30	1286.48	2217.165	1740.41
Closed	CM-ClaSP	1591.04	1611.44	1665.99	2158.76	2499.13	2955.19	Failed
Closed	CloFAST	2553.58	2456.77	2939.67	2326.90	2450.64	1880.79	Failed
Max	MaxSP	416.04	459.42	970.77	946.63	1075.76	Failed	Failed
Max	VMSP	652.35	788.27	736.09	362.55	645.86	637.58	Failed
Contiguous	VMSP (no gap)	100.95	1196.67	219.346	303.99	284.27	417.61	630.23
Contiguous	CM-SPAM (no gap)	112.96	112.36	205.11	284.65	533.012	756	1359.29

**Table 5:** Number of output patterns of Algorithms with different minimum support (min_sup) categorized by their constraints.

Constraint	Algorithm	*min_sup*						
		0.7	0.6	0.5	0.4	0.3	0.2	0.1
No	GSP	0	3	46	120	305	621	Failed
No	PrefixSpan	0	3	46	122	606	7967	Failed
No	CM-SPADE	0	3	46	122	606	7973	384,295
Closed	CM-ClaSP	0	3	46	122	606	7973	Failed
Closed	CloFAST	0	3	46	122	610	8068	Failed
Max	MaxSP	0	3	45	99	380	Failed	Failed
Max	VMSP	0	3	46	120	554	7627	Failed
Contiguous	VMSP (no gap)	0	3	46	120	305	620	1255
Contiguous	CM-SPAM (no gap)	0	3	46	120	305	633	1461

## Data Availability

Microsoft T-drive data used in this work can be obtained from: https://www.microsoft.com/en-us/research/publication/t-drive-trajectory-data-sample/.
